# Poly(lactic-*co*-glycolic acid) for reagent storage and controlled release in thermoplastic microfluidics

**DOI:** 10.1039/d5lc00632e

**Published:** 2026-01-13

**Authors:** Jaesung Lee, Evan H. Benke, Ian M. White, Don L. DeVoe

**Affiliations:** a Department of Mechanical Engineering, University of Maryland, College Park MD USA ddev@umd.edu; b Fischell Institute for Biomedical Devices, University of Maryland, College Park MD USA; c Fischell Department of Bioengineering, University of Maryland, College Park MD USA

## Abstract

The on-chip storage of dried reagents is an important technological challenge that must be addressed to improve the capabilities of microfluidic point-of-care (POC) chips. In this work, we investigate the use of poly (lactic-*co*-glycolic acid) (PLGA) as an encapsulant for the storage and controlled release of dried reagents integrated into disposable thermoplastic microfluidic chips. The PLGA layer allows multiple solid reagent deposits to remain isolated during sample introduction at room temperature and controllably released into the sample volume after heating the chip above a critical threshold temperature. Simple manual pipetting of a PLGA/ethyl acetate solution serves to form a protective PLGA shell encapsulating deposited reagents, with robust sealing between the PLGA and thermoplastic cyclic olefin polymer (COP) substrate preventing reagent leakage during sample introduction. When using a shell thickness below 20 μm to encapsulate nucleic acids as model reagents, over 90% of the deposits are retained following extended aqueous flow, while heating the chip above 40 °C leads to dramatic shrinkage of the PLGA, resulting in delamination of the encapsulating film and rapid reagent release. Using this approach, an on-chip loop-mediated isothermal amplification (LAMP) assay for the detection of methicillin-resistant *Staphylococcus aureus* (MRSA) is implemented using multiple encapsulated LAMP primer sets integrated directly into an array of on-chip wells. The PLGA encapsulation technique is shown to be a simple and effective method for reagent-integrated microfluidic device manufacturing, offering a new path towards true sample-in, answer-out point-of-care assays.

## Abstract 2

On-chip reagent storage and controlled release is demonstrated using poly(lactic-*co*-glycolic acid) (PLGA) as a polymer-based approach to reagent integration within thermoplastic microfluidic chips for nucleic acid amplification testing.

## Graphical abstract

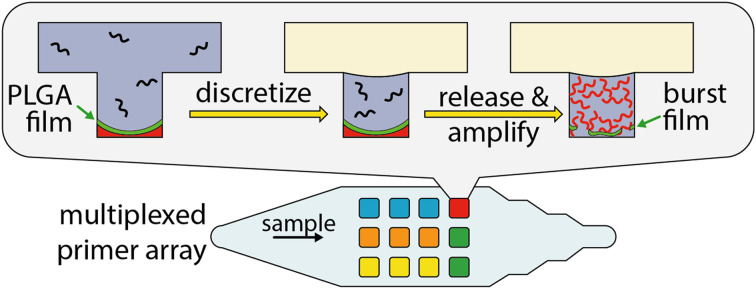

## Introduction

Microfluidic technologies have been widely leveraged for the development of clinical diagnostics, enabling rapid multiplexed analyses using sample volumes significantly smaller than conventional assays while providing sensitive, and actionable results for a range of disease states.^1–5^ While disposable microfluidic assays can offer important benefits for point-of-care testing, one of the fundamental limitations for many microfluidic diagnostics is the need for assay reagents to be introduced into the microfluidic devices prior to testing. This dependency can impose the need for additional instrumentation and operational steps, complicating device usage, increasing costs, limiting portability, and introducing barriers that can hinder the path to regulatory approval and clinical adoption. The need for addressing this constraint is particularly critical for the use of microfluidic assays in decentralized healthcare environments including hospital short turnaround testing labs, satellite labs, small clinics, and home settings where system size, cost, and complexity are determinative considerations for assay adoption.

The direct integration of reagents into microfluidic devices during device manufacture can serve to overcome this challenge, making it possible to realize the full potential of microfluidic technology for accessible point-of-care assays.^6^ By pre-loading reagents into disposable chips, assays can be performed without the need for external reagent storage or delivery, enabling fully self-contained diagnostic systems that can reduce operational complexity, simplify user interactions, and enhance reproducibility. Furthermore, because on-chip reagents can be resolubilized during sample or buffer introduction, they may be stored in a dried state to minimize degradation at elevated temperatures, extending the shelf life of the diagnostic device without the need for refrigerated storage. The incorporation of reagents into microfluidic chips thus holds great potential for expanding the reach of microfluidic assays and transforming near-patient diagnostics.

In the case of biomolecular assays, where precise control over the concentrations of assay components is often necessary, a central requirement for microfluidic reagent storage is that the reagents must be retained during sample introduction to ensure reliable control over their final concentrations within the on-chip reaction chambers. To this end, one approach to reagent integration involves storing deposited reagents within dedicated on-chip reservoirs, with valving used to isolate the reagents from the flow path used for sample introduction. Once the sample volumes are isolated, reagents are then introduced into the reaction chambers by opening the valves prior to assay initiation.^7,8^ While this approach can prevent reagent loss during sample loading, valving introduces unwanted complexity for both microfluidic chip design and system operation.

As an alternative to valving for physical compartmentalization, reagent encapsulation using hydrocarbon waxes has been employed to cover solid reagent deposits. This technique has the benefit of allowing reagents to be deposited directly within the desired reaction chambers, obviating the need to transport or otherwise manipulate reagents during the assay. Protecting the deposits with a water-insoluble shell of paraffin, petrolatum, or a purified alkane can enable aqueous sample to be introduced without disturbing the dried reagents, which can be solubilized following sample delivery by heating the system to melt the wax layer. The utility of paraffin wax-based reagent encapsulation has been demonstrated for various nucleic acid amplification tests based on polymerase chain reaction (PCR),^9^ loop mediated isothermal amplification (LAMP),^10^ and rolling circle amplification (RCA).^11^ While wax encapsulation can offer an effective method for controlled reagent release, paraffin is not compatible with some substrate materials commonly used for microfluidic devices. For example, although paraffin is insoluble with many thermoplastic materials including polycarbonate and acrylic, it is incompatible with other thermoplastics commonly used for microfluidic device fabrication including cyclic olefin polymers and co-polymers (COP and COC), where exposure to paraffin can result in significant polymer crazing and cracking. A more general limitation is that the wax layers must be deposited in a molten state, constraining the techniques available for material integration and complicating the deposition process. This can become a significant constraint as device size scales downward due to challenges with controlling the volume of molten wax delivered to microliter- or nanoliter-scale wells. The hydrophobic nature of hydrocarbon waxes can also prevent efficient sample filling and lead to bubble trapping within on-chip wells or microchannels.^12^

Rather than encapsulating reagents within an insoluble wax shell, an alternate approach is to embed reagents within a water-soluble polymer matrix before deposition within the microfluidic device. In this approach, the embedded reagents may be retained during sample filling while being fully solubilized following a short incubation period. The polymer can also serve to stabilize the reagents for long-term storage, extending their shelf life and relaxing the need for cold chain storage. Various natural or synthetic biocompatible polymers have been explored for reagent integration, with physical and chemical properties that can be tuned by selecting different molecular weights, mixing with other materials, or modifying chemical functional groups in the polymer branches. For example, agarose has been employed as a matrix for on-chip storage of nucleotides and polymerase to enable loop-mediate isothermal amplification (LAMP) reactions,^13,14^ and polyvinyl alcohol (PVA) has been used to maintain the viability of incorporated reagents for various microfluidic assays.^15,16^ Similarly, horseradish peroxidase (HRP) has been integrated into electrospun polyvinylpyrrolidone (PVP) nanofibers during synthesis for on-chip enzymatic assays.^17^ However, release rates of the reagent contained in these polymer matrices tend to be quite rapid, limiting their ability to retain reagents during sample filling. This is a particular constraint for microfluidic devices employing smaller reaction volumes, where complete loss or dispersion of reagents between multiplexed reaction chambers can be a significant challenge.

In this report, we describe a new reagent encapsulation technology based on poly (lactic-*co*-glycolic) acid (PLGA), a biodegradable polymer that has emerged as a popular encapsulant in the field of drug delivery due to its tunable drug release profile.^18^ When exposed to an aqueous environment, PLGA degradation occurs through ester bond hydrolysis over time scales that can be adjusted by changing the co-monomer ratio, with more rapid hydrolysis and greater hydrophilicity occurring at higher lactide content.^19,20^ Dissolution time scales can be on the order of days or weeks,^21^ making dissolution-based reagent release impractical for application to microfluidic assays. However, PLGA has been reported to exhibit rapid and significant shrinkage at elevated temperatures.^22,23^ We hypothesized that the slow degradation rate of PLGA could be harnessed to minimize the loss of encapsulated reagents during room-temperature sample introduction, with controlled release of reagents sequestered beneath the PLGA layer achieved by shrinking the polymer at elevated temperature, resulting in strain-induced rupture or delamination of the film from the chip surface. Here we test this hypothesis by forming PLGA-coated deposits of DNA oligomers in an array of microwells patterned in a cyclic olefin polymer (COP) chip. The PLGA films were found to retain more than 90% of the deposited DNA after 10 min of exposure to water at room temperature, with rapid release of the reagents achieved after increasing the chip temperature above 40 °C. Using this PLGA encapsulation technique, a multiplexed on-chip LAMP assay was successfully demonstrated for the detection of methicillin-resistant *Staphylococcus aureus* (MRSA). The PLGA-coated LAMP primers showed successful amplification after 30 days of room temperature storage with minimal increase in time-to-positive (TTP) values for the MRSA assay. This novel polymer-based encapsulation method opens a new route to manufacturable self-contained microfluidic chips for point-of-care diagnostics.

## Materials and methods

### Microfluidic chip fabrication

A 4 × 3 array of square wells, each with side length of 1.8 mm, depth of 1.4 mm, and center-to-center spacing of 2.7 mm for a nominal well volume of 4.5 μL, was formed in a 2 mm thick COP layer (1420R COP wafer, Microfluidic ChipShop) by CNC machining using an 800 μm diameter end mill. After well fabrication, the COP surface was hydrophilized by oxygen plasma activation (PE-25, Plasma Etch, Inc.) followed by incubation in a fresh Pluronic F-108/deionized water solution (20 mg mL^−1^) for 1 h. The well plate was blown dry with filtered air and heated on a hot plate at 70 °C for 30 min. The dried well plate was washed with deionized water and stored in a dry environment until its use. In addition to the COP well plate, an acrylic plate (1.0 mm thick) was cut into a channel pattern using a laser cutter (Fusion M2, Epilog Laser) to define a flow cell comprising an open chamber above the microwell array and inlet and outlet channels on opposing sides of the chamber. Next, 0.65 mm diameter holes were drilled into a mating COP cover plate to form fluidic access ports matched to channel inlet and outlet. The COP well layer, acrylic channel layer, and COP cover layer were bonded together using 25 μm thick double-sided tape (468MP, 3 M) patterned using a cutter plotter (Cameo 4, Silhouette), for a total channel height of 1.05 mm. Stainless steel 22 gauge needle segments (Hamilton Syringe) were inserted into the inlet and outlet ports to provide tubing connections.

### Reagent deposition and PLGA coating

A mixture of DNA primers and trehalose (d-(+)-trehalose dihydrate, Sigma Aldrich) was integrated in the chips for the LAMP assay. Detailed information of the LAMP assay components can be found in Tables S1–S4. After evaluating multiple compositions of PLGA (Polysciences, Inc.) for LAMP compatibility (Fig. S1), an ester-terminated PLGA with 90 : 10 lactic : glycolic acid was employed for all further experiments. As the first step of reagent integration, a 1.6 μL volume of the primer/trehalose solution was manually pipetted into each COP well and dried under vacuum at −27.5 inHg for 2 h, followed by overnight evacuation at −29.5 inHg. After drying, a 2.4 μL droplet of 2.0% PLGA (90 : 10, molecular weight ∼<10 k, Polysciences, Inc.)/ethyl acetate (Sigma Aldrich) solution was manually pipetted into the well. The PLGA droplet was dried under vacuum at −24 inHg for 2 min, −26.5 inHg for 3 min, and −29.5 inHg for 10 min. Two cycles of the PLGA coating process were conducted to form the final PLGA layers. After PLGA coating, 2.6 μL of SPAN20 in isopropyl alcohol (0.05 v/v%) was dispensed in the COP wells to encourage the filling of the wells with aqueous solutions. When measuring the thickness of the PLGA layer, DiI dye (∼1 mg mL^−1^) was mixed with the PLGA/ethyl acetate solution to enable cross-sectional imaging of the PLGA film by confocal fluorescence microscopy.

### Release rate measurements

DNA release rate measurements were performed by depositing a droplet of fluorescein amidite (FAM)-conjugated DNA (20 nt long, Integrated DNA Technologies, Inc.) mixed with trehalose on a planar COP surface. The droplet was dried and encapsulated with PLGA using the same methods employed for the reagent integration in wells. The COP substrate was capped with a flow cell identical to the microchannel design used for the well chips. The substrate was mounted on a heating plate of the custom-built thermocycler with a fluorescence microscope used to monitor fluorescence in the encapsulated reagent deposits over time. Deionized water was continually perfused through the system at 50 μL min^−1^ during all tests to remove released DNA from the optical detection region to minimize background signal.

### LAMP assay

To evaluate the reagent encapsulation and release process for on-chip LAMP, a multiplex assay for MRSA was implemented in a COP chip containing 12 wells, with 3 wells each containing LAMP primers targeting the *nuc*, *mecA,* and *femB* genes, 2 wells containing both the *mecA* primer set and MRSA genomic DNA (10^2^ cp μL^−1^) as positive controls, and 1 well without primers as a negative control. Details of the LAMP components are provided in Table S1. The primer sequences were sourced from previous studies.^24–26^ To implement the on-chip LAMP assay, a syringe pump introduced 300 μL of LAMP buffer containing polymerase and genomic DNA from either MRSA or methicillin-susceptible *S. aureus* (MSSA) through Tygon tubing connected to the microfluidic chip inlet port at a flow rate of 400 μL min^−1^, filling the wells with the DNA solution. Silicone oil was then injected to discretize the aqueous phase into individual wells. The priming and partitioning steps were completed within approximately 2 min. After filling, the tubing was disconnected, and the chip was placed on the heater of the custom thermocycler. The temperature of the heater was ramped from room temperature to 61 °C and maintained at 61 °C for at least 60 min. Fluorescence images were captured every 10 s, and the average fluorescence intensity for each well was quantified using ImageJ software.

## Results and discussion

### Reagent integration using PLGA in a cyclic olefin polymer (COP) well

**Fig. 1:**
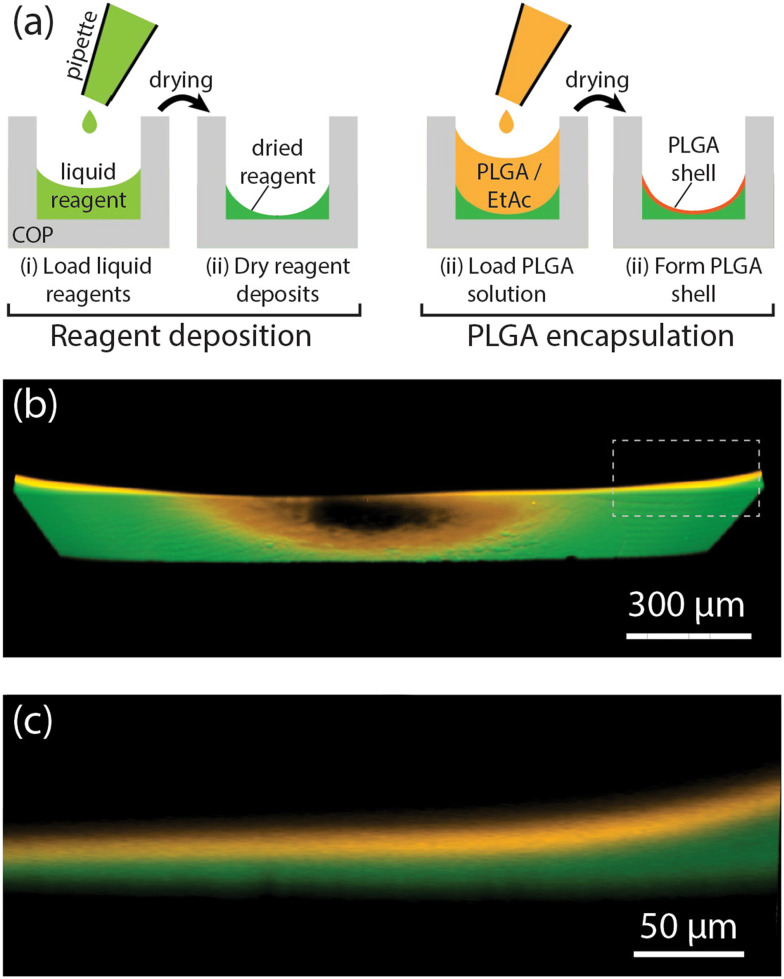
Deposition of reagents and PLGA film formation in a COP microwell. (a) Overview of the reagent integration process. (b) Confocal fluorescence image showing an oblique perspective view looking up into the central area of the base of COP microwell (1.8 mm side length), showing a PLGA layer (orange) covering a deposit of fluorescein-conjugated DNA (green). The dark region in the center reflects the tendency for reagents to migrate to the well sidewalls due to surface tension. (c) Side view of the boxed region in panel (b) revealing the PLGA layer thickness.

The reagent integration process combines pipetting and drying of reagent solutions within a microliter-scale COP well followed by encapsulation of the dried reagents with a thin layer of PLGA, as outlined in Fig. 1a. First, a droplet of aqueous reagent solution is dispensed into a well, with subsequent vacuum drying resulting in a solid deposit at the well bottom. As water evaporates from the deposited volume, the fluid remains pinned to the outer edge of the well base due to surface tension, resulting in a concave deposit of dried reagents concentrated along the bottom well perimeter. After the reagents have fully dried, an encapsulating PLGA layer is formed by manually pipetting PLGA/ethyl acetate solution into the well and allowing the solvent to evaporate to form a solid PLGA film. The distribution of PLGA and encapsulated DNA within the middle region of a well can be seen in the confocal fluorescence images in Fig. 1b and c. The reagent solution becomes pinned at the rim of the well base due to capillary effects, with the formation of a curved interface reducing free energy of the system. As the solution dries, the remaining liquid becomes isolated around at the corners between the well base and walls, concentrating the reagents to that region and forming a continuous ring of dried deposits after complete evaporation of the carrier. The thickness of the PLGA layer can be tuned by adjusting the volume and concentration of the PLGA solution and the number of repeated PLGA deposition cycles. For the selected PLGA solution conditions (2.4 μL volume of 2.0% PLGA), two cycles of the PLGA coating process resulted in an encapsulation layer with an average thickness of approximately 18 μm.

### Temperature-dependent release of PLGA-encapsulated reagents

Unlike reagent integration techniques employing paraffin wax, where reagent release occurs by melting the wax layer, the release of PLGA-encapsulated reagents relies on temperature-controlled shrinkage and bursting of the polymer film rather than melting or dissolution. The volumetric shrinkage of PLGA at elevated temperatures occurs due to polymer chain relaxation as previously reported for electrospun PLGA membranes,^22,23^ with the degree of volume change increasing with incubation temperature and decreasing with the PLGA glass transition temperature.^22^ Prior to film shrinkage, transport of reagent molecules through the PLGA layer can occur by diffusion or osmotic pumping within the hydrated polymer pores, or following leakage after hydrolysis and mechanical degradation of the polymer structure.^27^ At room temperature, the PLGA polymer network is sufficiently dense to prevent rapid diffusion or osmotic transport of DNA through the pores, while slow low-temperature polymer erosion rate of PLGA similarly prohibits loss of encapsulated reagent over relevant assay time scales. As the temperature begins to increase, thermal shrinkage increases the density of the polymer network and reduces pore dimensions. As a result, reduced diffusion rates and reagent leakage is expected at moderate temperatures. However, as the temperature increases, tensile strain within the polymer film ultimately leads to film rupture or substrate delamination, resulting in rapid burst release of encapsulated reagents. Because the majority of the reagents are packaged as a contiguous deposit, once a portion is exposed to water the entire deposit is quickly dissolved, while thermally-driven convection induces rapid mixing within the full well volume.

**Fig. 2:**
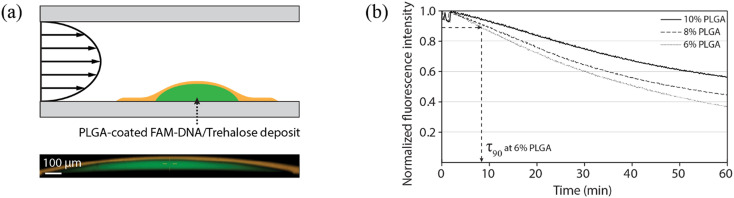
Dependence of the release rate on PLGA layer thickness and temperature. (a) (Top) Schematic of the measurement system, and (bottom) a confocal microcopy cross-section showing the PLGA (orange)-encapsulated FAM-DNA (green)/trehalose deposit on a flat COP substrate. The PLGA layer was mixed with DiI for the visualization. (b) Temporal change of fluorescence from an encapsulated DNA deposit at room temperature for various concentrations of PLGA solution.

The reagent release process was characterized using a microfluidic flow cell with planar deposits of fluorescein-conjugated DNA/trehalose (with typical dimensions around 1.2 mm diameter and 75 μm thickness) encapsulated within a layer of PLGA (Fig. 2a). Release rates were determined by monitoring average fluorescence levels of the deposit, with reagent loss leading to a monotonic decrease in fluorescence. Reagent loss was quantified by the measured time for the fluorescence intensity to reach 90% of the initial value (*τ*_90_). To evaluate the impact of polymer film thickness on reagent leakage at room temperature, measurements were performed using PLGA films prepared with different polymer concentrations (Fig. 2b). The correlation between the polymer concentrations and the polymer layer thicknesses is provided in Fig. S2. As anticipated, thicker films resulted in slower reagent loss, with measured *τ*_90_ values at room temperature increasing from 8.4 min to 13.2 min as film thicknesses increased from 14.9 μm to 18.6 μm.

**Fig. 3:**
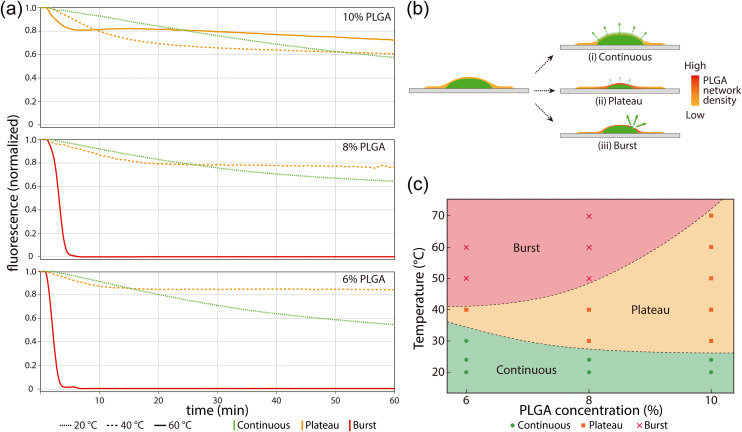
(a) Typical release curves of PLGA-encapsulated fluorescent DNA deposits at varying temperatures and initial PLGA solution concentrations. (b) Three different release modes observed for PLGA-encapsulated DNA: (i) continuous, (ii) plateau, and (iii) burst. (c) Experimental release mode regimes mapped over a range of incubation temperatures and PLGA concentrations.

We further evaluated temperature-dependent release rates by monitoring reagent release over a period of 60 min with varying PLGA concentration at temperatures up to 70 °C (Fig. 3a). Extracted fluorescence curves from these experiments (Fig. 3b and c) reveal three distinct release modes defined by a continuous release regime, plateaued release regime, and a rapid burst regime. Continuous release is seen to occur at low temperatures below approximately 40 °C for all polymer concentrations. Within an intermediate temperature range between 40–50 °C, reagent loss initially occurs at a higher rate compared with the continuous regime, but quickly plateaus with little or no additional reagent loss over the remainder of the testing period. As discussed above, this plateau behavior is believed to result from shrinkage of the polymer matrix, leading to a reduction in pore dimensions that inhibits the transport through the PLGA film. At higher temperatures in the range of 50–60 °C, the thinner PLGA layers rupture as their tensile stress begins to exceed the mechanical strength of the films, resulting in rapid reagent release and dissolution within the surrounding aqueous buffer (Movie S1). As seen in Fig. 3c, the burst temperature depends on the film thickness, with films prepared at higher polymer concentrations exhibiting greater mechanical resistance to shrinkage-induced rupture. Film rupture, which takes place at temperatures above the burst regime threshold, was observed to occur solely within the region of the PLGA layer located above the dried reagent deposit, with the portion attached to the COP substrate remaining intact and well-bonded to the thermoplastic surface. When employing PGLA layers deposited within on-chip microwells, the bursting process tended to result in detachment of PLGA from the well sidewalls rather than direct tearing or rupture of the film (Fig. S4) likely due to the smaller contact surface for the in-well tests compared with experiments performed using planar deposits.

One surprising observation from these experiments is that the room temperature release rates are significantly faster than the days-to-weeks scale rates seen in typical drug-loaded PLGA capsules or particles. In the absence of film shrinkage and rupture, we considered nanocracks induced by osmotic pressure^28^ as a potential mechanism to explain this behavior, but this hypothesis was ruled out because it could not explain the plateaued release mode observed at moderate temperatures; if nanoscale cracks had formed in the film, temperature elevation would be expected to widen the cracks and cause an increase in release rate, rather than a decrease. Instead, we hypothesize that the unusually high release rate can be attributed to a combination of rapid swelling of the PLGA layer and large free volume in the PLGA network. First, because the PLGA used in this work (∼10 kDa molecular weight) is below the critical threshold (∼20 kDa) for rapid swelling,^29,30^ the resulting polymer layer is expected to swell quickly after hydration. Second, the processing conditions used for PLGA film formation are known to have a significant impact on drug release profile.^31–33^ In our case, the fabrication process for the encapsulating PLGA layer involves exceptionally rapid solvent evaporation in a vacuum environment, with ethyl acetate removal occurring within a few seconds. This rapid evaporation process likely results in a PLGA network with lower density and larger free volume than typical films formed by gradual polymer precipitation during evaporation of solvent from a PLGA emulsion. The large free volume of the resulting network not only has the potential to form channels for encapsulated DNA to escape through diffusion and osmotic pumping, but also further enhances the polymer swelling rate.

### Multiplexed LAMP assay using integrated reagents

**Fig. 4:**
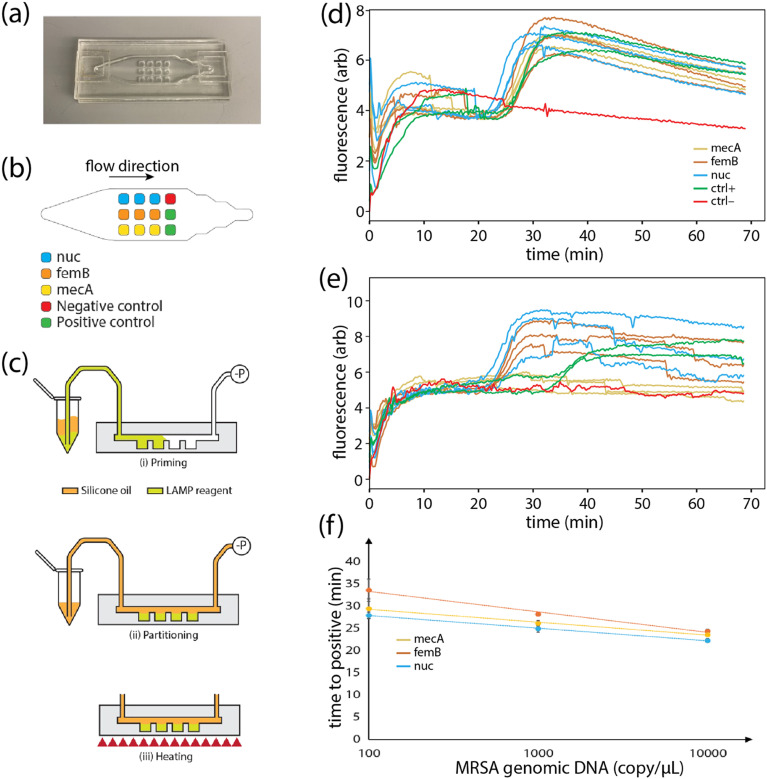
Multiplexed LAMP assay for MRSA detection. (a) Photo of the 12 well COP chip. (b) Primer configuration within the well array. (c) Assay operation procedure. Amplification curves for each of the 3 target genes and ± controls for chip run using (d) MRSA and (e) MSSA samples at a concentration of 10^4^ cp μL^−1^. Each curve corresponds to a single well. No amplification of *mecA* was observed for MSSA, enabling the differentiation from MRSA. Fluorescence fluctuations reflect air bubble generation and escape. (f) Time-to-positive values for each target across a 3 log_10_ concentration range for MRSA genomic DNA samples (*N* = 3).

To evaluate the performance of the PLGA-based reagent integration technique, we fabricated a set of microfluidic COP chips containing an array of 12 independent wells located beneath a sample delivery channel to implement a multiplexed LAMP assay for MRSA detection (Fig. 4a). Gene-specific LAMP primers were first pipetted into the wells, with independent sets of 3 wells containing primers targeting the heat-resistant nuclease (nuc) gene and femB gene associated with peptitoglycan cell wall synthesis as markers of *S. aureus*, together with the mecA gene associated with resistance to methicillin and other β-lactam antibiotics (Fig. 4b), allowing the resulting assay to differentiate MRSA from methicillin-susceptible *S. aureus* (MSSA) which typically lacks the mecA gene. After primer deposition and PLGA encapsulation layer formation, the wells were primed with LAMP buffer containing polymerase together with either MRSA or MSSA genomic DNA, followed by partitioning with silicone oil (Fig. 4c). The PLGA film thickness used in these experiments was approximately 18 μm, corresponding to a *τ*_90_ value (Fig. 2) of 13.2 min. To confirm that minimal reagent loss occurs for sample filling times shorter than *τ*_90,_ on-chip LAMP assays were performed using well priming flow rates ranging from 50–400 μL min^−1^, corresponding to filling times between 0.75–6.0 min. As expected, no appreciable change in time-to-positive values or false positives were observed within this range. To minimize total assay time, the highest flow rate (400 μL min^−1^) was used for all further LAMP assay tests.

The in-well temperature during LAMP was estimated by measuring the temperature of the bottom heater and the top chip surface over a range of heater temperature set points, and employing Fourier's law for one-dimensional heat conduction to estimate the internal temperature distribution within the chip. Using this approach, and targeting a reaction temperature of 60 °C, an optimal temperature set point of 63 °C was predicted. However, a series of experiments performed over a heater temperature range between 60–65 °C revealed that slightly lower heater set points resulted in shorter TTP values (Fig. S5). Based on these experiments, a temperature set point of 61 °C was used for all LAMP assays.

During the on-chip LAMP process, an unexpected nonlinear increase in fluorescence intensity was observed at the beginning of thermal incubation (Fig. 4d and e and S3). To identify the source of this non-specific fluorescence signal, which was absent when performing identical on-chip amplification tests in the absence of PLGA (data not shown), we measured the fluorescence increase in various combinations of the LAMP components (Note S1). The results revealed that non-specific fluorescence was generated only when incubating the PLGA layer with either primers or polymerase, with a more pronounced effect observed with primers. This result indicates that the non-specific fluorescence likely arises from adsorption or preferential residence of the DNA-intercalating dye within the PLGA layer, with conformational change of the dye molecules facilitated by the presence of DNA or polymerase. Shrinkage of the PLGA layer may also increase the dye density within the polymer, making the fluorescence readily detectable at the start of heating. Because the resulting non-specific background fluorescence was significantly lower than the fluorescence signal due to target amplification, it was not found to impede assay readout (Fig. S3).

Reliable MRSA detection was observed using the primer-integrated COP chips, enabling MRSA to be efficiently differentiated from MSSA within a 60 min incubation period (Fig. 4d and e). The initial 10 min period for each amplification curve in Fig. 4 is omitted to improve data interpretation following baseline normalization. Time-to-positive (TTP) values for the LAMP assay (Fig. 4f) exhibited a log-linear relationship for DNA concentration in the range from 10^2^–10^4^ cp μL^−1^. However, at 1 cp μL^−1^ and 10 cp μL^−1^, not all wells exhibited amplification, with 74% of wells containing primers reporting amplification at 10 cp μL^−1^, and only 4% of reporting amplification at 1 cp μL^−1^. The 10^2^ cp μL^−1^ detection limit is believed to be due to loss of genomic DNA *via* non-specific adsorption within the sample delivery tubing, rather than an innate limitation of the reagent integration process, as similar results were observed during on-chip LAMP without PLGA encapsulation (data not shown). Surface passivation of the tubing used to connect the chip to the sample injection reservoir may help further extend the lower limit of detection.

### Room temperature storage of integrated LAMP primers

**Fig. 5:**
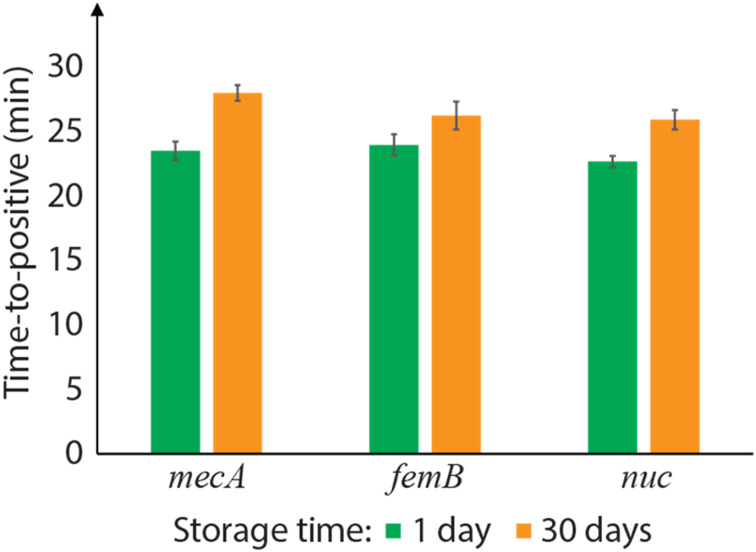
Time-to-positive (TTP) values for on-chip LAMP assays performed in primer-integrated wells after 1 day and 30 day of room temperature storage (10^4^ cp μL^−1^ MRSA genomic DNA; *N* = 3).

To evaluate the stability of integrated primers over time, a set of well array chips with PLGA-encapsulated DNA primer/trehalose were stored at room temperature for 30 days. On-chip LAMP in those stored chips showed successful amplification, but with TTP values that were on average 15% longer than the chips stored for 1 day (Fig. 5). Longer storage periods were not tested in this work. One possible reason for the delayed time-to-positive values is a reduction of the release rate due to physical aging of the PLGA layer. PLGA films formed by rapid solvent evaporation are initially in a high energy state, and the material trends toward thermodynamic equilibrium *via* polymer chain relaxation at ambient conditions.^33^ The polymer chain relaxation leads to a denser PLGA network and changes the film strain state, which may retard the burst release process. This effect of physical aging on the release rate is a topic of future study. Another possible cause for the TTP increase is natural primer degradation within the trehalose matrix or the inhibition caused by carboxylic acids generated through PLGA degradation mediated by moisture. PLGA is hygroscopic, and its water-induced degradation produces acidic terminal groups in its chain, which may negatively impact the LAMP performance. Although the hydrolytic degradation of PLGA in ambient condition is slow and expected be negligible over several months,^34,35^ the effect could become significant over longer storage periods. To address this potential concern for longer-term storage of the disposable diagnostic chips, vacuum sealing or nitrogen-purged packaging may be desirable to minimize degradation.^31^

## Conclusions

In this report, we demonstrated the use of PLGA as a novel encapsulant for on-chip reagent integration in thermoplastic COP microwells. The technique is easy to implement and allows tunable reagent release rates, with 20 μm thick PLGA layers shown to retain over 90% of the deposited DNA for over 20 min after exposure to aqueous solution at room temperature, ensuring sufficient retention of reagents during sample filling, while enabling controlled release at elevated temperatures. Thermal shrinkage of the PLGA layer at temperatures above 40 °C enabled the burst release of encapsulated DNA/trehalose deposits, with over 80% release achieved within 6 min under an aqueous flow at 60 °C. Leveraging this temperature-dependent release behavior, we successfully demonstrated the reagent encapsulation method through on-chip LAMP assays for MRSA detection using primer-integrated COP wells. Compared with established methods for reagent integration and controlled release using paraffin wax, the PLGA-based reagent integration process can expand the range of compatible substrate materials while improving reagent encapsulation and release at smaller well size scales. The presented approach expands the options for integrating reagents into microfluidic point-of-care devices, and highlights the potential of new polymer-based coatings for enhancing the integration of reagents directly into manufacturable microfluidic assays.

## Conflicts of interest

There are no conflicts to declare.

## Supplementary Material

LC-026-D5LC00632E-s001

LC-026-D5LC00632E-s002

## Data Availability

The data underlying the presented results are available within the article and its supplementary information (SI). Supplementary information is available. See DOI: https://doi.org/10.1039/d5lc00632e.
