# Supplementary material for: Poly(lactic-co-glycolic acid) for reagent storage and controlled release in thermoplastic microfluidics
Source: Lab Chip. 2026 Jan 13;26(4):897–905. doi: 10.1039/d5lc00632e (PMC12810686; doi:10.1039/d5lc00632e)
Supplement: LC-026-D5LC00632E-s002 [file LC-026-D5LC00632E-s002.pdf]

Supplementary Information (SI) for Lab on a Chip.
This journal is © The Royal Society of Chemistry 2026

Supplementary Information

Poly(lactic-*co*-glycolic acid) for Reagent Storage and Controlled Release in Thermoplastic Microfluidics

Jaesung Lee,[a,b] Evan H. Benke,[b,c] Ian M. White,[b,c] and Don L. DeVoe *[a,b,c]

[a]Department of Mechanical Engineering, University of Maryland, College Park MD
[b]Fischell Institute for Biomedical Devices, University of Maryland, College Park MD
[c]Fischell Department of Bioengineering, University of Maryland, College Park MD

Table S1: LAMP primer target sequences (nuc and mecA primer sequences from Zhao 2022, and femB primer sequences from Chen 2017 and Nanayakkara 2019).

Primer	Sequence (5' → 3')
nuc F3	CGATTGATGGTGATACGGTTA
nuc B3	CACGTCCATATTTATCAGTTCT
nuc FIP	TCTCTACACCTTTTTTAGGATGCTTGTCAAACAATGACATTCAGACT
nuc BIP	TATGGTCCTGAAGCAAGTGCACCTTTGTCAAACTCGACTTC
nuc Loop B	TACGAAAAAAATGGTAGAAAATGCA
mecA F3	TGATGCTAAAGTTCAAAAGAG
mecA B3	GTAATCTGGAACTTGTTGAG
mecA FIP	TGAAGGTGTGCTTACAAGTGCTAATCAACATGAAAAATGATTATGGCTC
mecA BIP	TGACGTCTATCCATTTATGTATGGCAGGTTCTTTTTTATCTTCGGTTA
mecA Loop F	TCACCTGTTTGAGGGTGG
femB F3	TGTTTAAATCACATGGTTACGAG
femB B3	TCACGTTCAAGGAATCTGA
femB FIP	TACCTTCAAGGTTTAATACGCCCATCATCATGGCTTTACAACTGAG
femB BIP	ACACCCGAAACATTGAAAAAGACACTTTAACACCATAGTTTATCGCTT
femB Loop F	TCGTACTTGGCTCGATGTATCATA

Table S2: Lamp reaction buffer composition (20 µL/reaction).

Reagent	Concentration
Trehalose	10% wt
Isothermal amplification buffer (https://www.neb.com/en-us/products/b0537-isothermal-amplification-buffer)	1x
dNTPs (https://www.neb.com/en-us/products/n0447-deoxynucleotide-dntp-solution-mix)	0.6 mM
EvaGreen dye (https://biotium.com/product/evagreen-dye-20x-in-water/)	1x
F3 primer (IDT)	0.2 µM
B3 primer (IDT)	0.2 µM
FIP primer (IDT)	1.6 µM

BIP primer (IDT)	1.6 µM
Loop primer (IDT)	0.8 µM
BST 2.0 WarmStart DNA polymerase (glycerol free) (New England Biolabs)	6.4 U

Table S3: Composition of on-chip primer deposits for LAMP assays (1.7 µL/reaction, 2.6x concentrated).

**Reagent**	**Concentration**
Trehalose	26% wt
F3 primer (IDT)	0.52 µM
B3 primer (IDT)	0.52 µM
FIP primer (IDT)	4.16 µM
BIP primer (IDT)	4.16 µM
Loop primer (IDT)	2.08 µM

Table S4: On-chip LAMP solution (300 µL/chip)

**Reagent**	**Concentration**
BST 2.0 WarmStart DNA polymerase (glycerol free) (New England Biolabs)	96 U
Isothermal amplification buffer	1x
dNTPs	0.6 mM
EvaGreen dye	1x

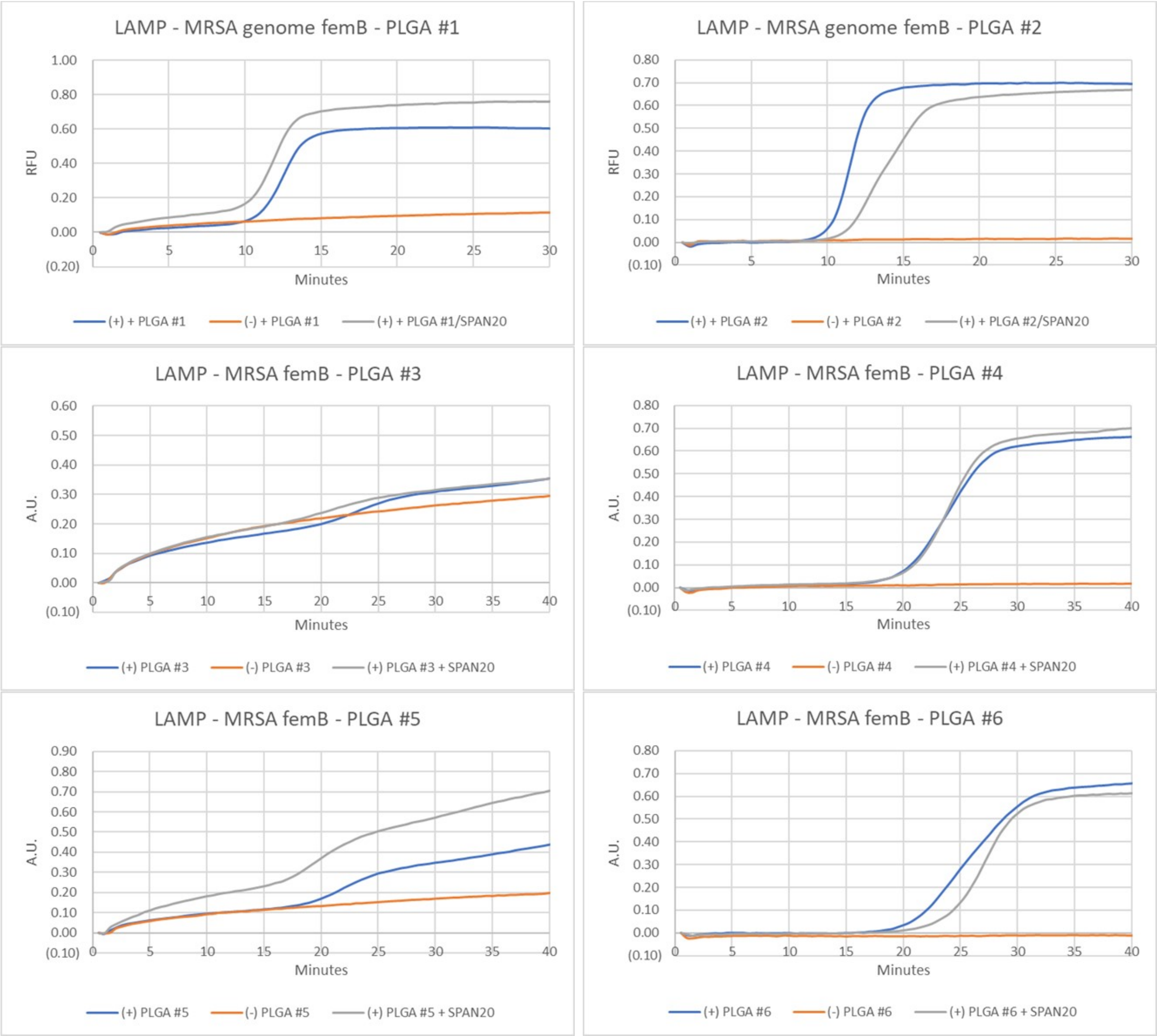

Figure S1: Six different PLGA compositions were evaluated for potential LAMP inhibition. Primers were deposited at the base of an Eppendorf tube and encapsulated by a PLGA layer via PLGA/acetone solution dispensing and drying. LAMP master mix and MRSA genomic DNA were added to the tube and incubated at 60 ^{o}C in a benchtop thermal cycler (Roche LC480). PLGA compositions #1, #2, #4, and #6 terminated with an ester (-COOR), while #3 and #5 terminated with carboxylic acid (-COOH). No inhibition was observed for the ester-terminated PLGAs, while the carboxyl-terminated PLGAs resulted in significant inhibition. Based on these results, an ester-terminated PLGA (90:10, MW≤10k) was used for all further encapsulation and LAMP experiments.

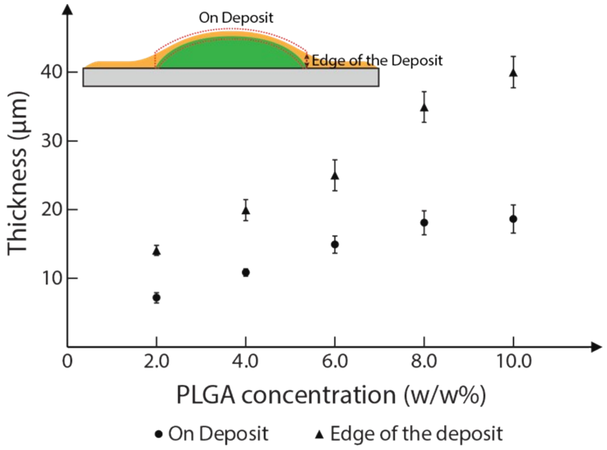

Figure S2: Experimental relationship between PLGA concentration in the initial ethyl acetate solution and final PLGA layer thickness measured at the center and edge of the dried reagent deposit.

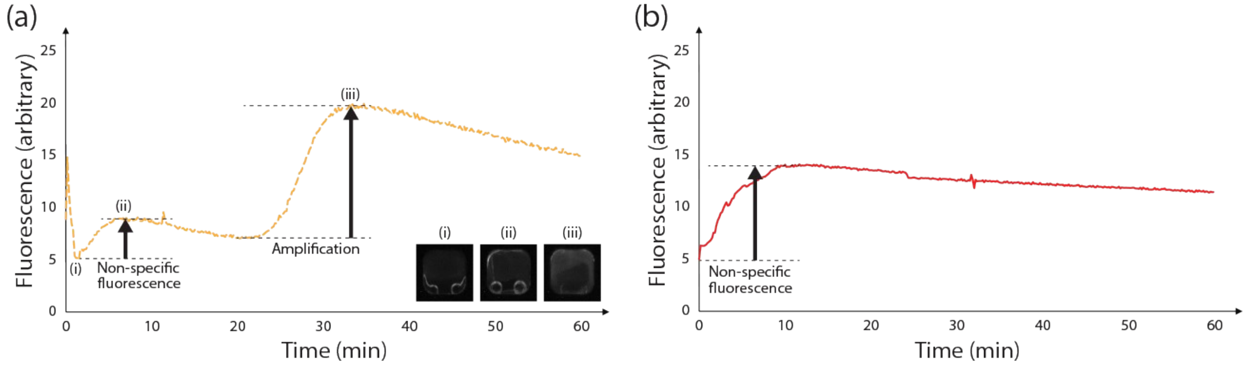

Figure S3: (a) On-chip LAMP amplification curve from single well containing PLGA-encapsulated *mecA* primers. A characteristic two-step response is observed, with an early non-specific fluorescence response followed by an increase due to primer-mediated amplification. The steep decrease of fluorescence near t = 0 is due to the inherent temperature dependence of EvaGreen fluorescence. Fluorescence images of a *mecA* well of three different time points: (i) the beginning of the incubation, (ii) after non-specific fluorescence was generated, and (iii) after amplification occurred were included. (b) Amplification curve for a negative control, with only a non-specific fluorescence increase observed.

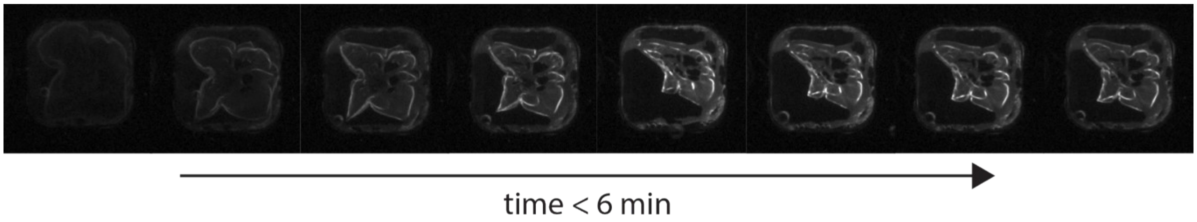

Figure S4: Time-lapse images of a shrinking PLGA layer in a well upon heating at 61 ^{o}C. The fluorescence of the PLGA film in the well increases non-specifically upon the start of thermal incubation. The brightness of the photos were enhanced for visibility.

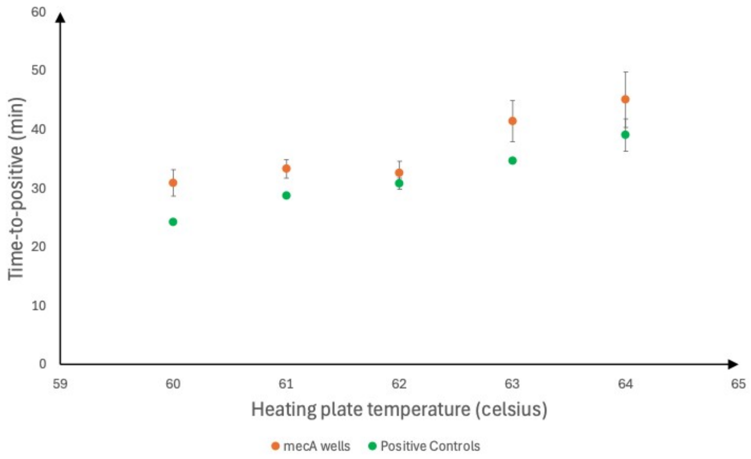

Figure S5: Time-to-positive (TTP) of primer-integrated on-chip LAMP assays for *mecA* and positive controls at various heating plate temperatures.

Note S1: Non-specific fluorescence was observed from the PLGA layer during on-chip LAMP (Figure S2) that was absent in the release rate experiments. To identify the source of this non-specific signal, a set of on-chip reactions were performed at 61 °C using various combinations of LAMP components deposited in the wells and encapsulated with PLGA (✓ = reagent included, X = reagent excluded):

Test #	PLGA	Primers	Polymerase	dNTP	LAMP buffer	EvaGreen	Fluorescence intensity
1	✓	✓	✓	✓	✓	✓	High
2	✓	✓	✓	✓	✓	X	Low
3	✓	✓	✓	✓	X	✓	High
4	✓	✓	✓	X	X	✓	High
5	✓	X	✓	X	X	✓	Medium
6	✓	X	X	X	X	✓	Medium
7	✓	X	X	X	X	X	Low

The measured fluorescence intensity was found to increase in the following order: #2,7 < 5,6 < 1,3,4. Without EvaGreen, the PLGA layer did not generate a meaningful level of fluorescence (tests #2 and 7). A strong increase in non-specific fluorescence occurred only in the presence of both polymerase and primers. We hypothesize that the non-specific fluorescence during the early stage thermal incubation may be caused by preferential residence of EvaGreen in the PLGA layer with help from nucleic acids and polymerase, leading to a reduction in vibrational and rotational freedom of the intercalating dye, and thus an increase in its fluorescence, due to thermal shrinkage of PLGA.

Movie S1: Video showing burst release of fluorescein-labeled DNA encapsulated with PLGA upon heating at 60 °C (1 second movie time corresponds to 1 min real time). The concentration of PLGA/ethyl acetate solution used to form the PLGA layer was 8.0%.
